# Supplementary material for: Evaluating the effect of metabolic traits on oral and oropharyngeal cancer risk using Mendelian randomization
Source: eLife. 2023 Apr 12;12:e82674. doi: 10.7554/eLife.82674 (PMC10147379; doi:10.7554/eLife.82674)
Supplement: Supplementary file 2. — Supplementary file 2, Table 2A. Assessing weak instrument bias (F-statistic) and proportion of variance in the phenotype (R2) explained by metabolic phenotype instruments. Abbreviations: BMI, body mass index; WC, waist circumference; WHR, waist–hip ratio; T2D, type 2 diabetes mellitus; HbA1c, glycated haemoglobin; FG, fasting glucose; FI, fasting insulin; SBP, systolic blood pressure; DBP, diastolic blood pressure. Supplementary file 2, Table 2B. Assessing heterogeneity of single nucleotide polymorphism effect estimates in inverse variance weighted (IVW) and MR-Egger regression for metabolic disorder analysis. Abbreviations: Q, Q-statistic; df, degrees of freedom; p, p-value; BMI, body mass index; WC, waist circumference; WHR, waist–hip ratio; T2D, type 2 diabetes mellitus; HbA1c, glycated haemoglobin; FG, fasting glucose; FI, fasting insulin; SBP, systolic blood pressure; DBP, diastolic blood pressure. Supplementary file 2, Table 2C. Assessing directional pleiotropy through MR-Egger intercept for metabolic disorder analysis. Abbreviations: SE, standard error; p, p-value; BMI, body mass index; WC, waist circumference; WHR, waist–hip ratio; T2D, type 2 diabetes mellitus; HbA1c, glycated haemoglobin; FG, fasting glucose; FI, fasting insulin; SBP, systolic blood pressure; DBP, diastolic blood pressure. Supplementary file 2, Table 2D. MR-PRESSO outliers detected results in the analysis of metabolic disorders on combined oral and oropharyngeal cancer risk. Abbreviations: Q-stat, Cochran’s Q-statistic; BMI, body mass index; WC, waist circumference; WHR, waist–hip ratio; T2D, type 2 diabetes mellitus; HbA1c, glycated haemoglobin; FG, fasting glucose; FI, fasting insulin; SBP, systolic blood pressure; DBP, diastolic blood pressure. Supplementary file 2, Table 2E. MR-PRESSO results for metabolic disorders on combined oral and oropharyngeal cancer. Abbreviations: SE, standard error; p, p-value; BMI, body mass index; WC, waist circumference; WHR, waist–hip ratio; T2D, type 2 d [file elife-82674-supp2.docx]

**Supplementary file 2**

**Supplementary Tables 2A through 2L**

**Supplementary file 2, Table 2A.** Assessing weak instrument bias (F-statistic) and proportion of variance in the phenotype (*R*^2^) explained by metabolic phenotype instruments

|  | R^2^ | F-statistic |
| --- | --- | --- |
| **BMI** | 0.040 (4.0%) | 89.5 |
| **WC** | 0.012 (1.2%) | 60.1 |
| **WHR** | 0.023 (2.3%) | 76.1 |
| **T2D** | 0.022 (2.2%) | 113.7 |
| **HbA_1c_** | 0.018 (1.8%) | 70.4 |
| **FG** | 0.030 (3%) | 133.6 |
| **FI** | 0.005 (0.5%) | 33.3 |
| **SBP** | 0.008 (0.6%) | 56.9 |
| **DBP** | 0.006 (0.6%) | 56.5 |

Abbreviations: BMI, body mass index; WC, waist circumference; WHR, waist-hip ratio; T2D, type 2 diabetes mellitus; HbA_1c_, glycated haemoglobin; FG, fasting glucose; FI, fasting insulin; SBP, systolic blood pressure; DBP, diastolic blood pressure.

**Supplementary file 2, Table 2B.** Assessing heterogeneity of single nucleotide polymorphism effect estimates in inverse-variance weighted (IVW) and MR Egger regression for metabolic disorder analysis.

| **Exposure** | **Exposure dataset** | **Q IVW** | **df** | **P** | **Q MR Egger** | **df** | **P** |
| --- | --- | --- | --- | --- | --- | --- | --- |
| BMI | Pulit *et al.* GWAS ^1^ | 311.47 | 271 | 0.05 | 309.67 | 270 | 0.05 |
| WC | Pulit *et al.* GWAS ^1^ | 50.35 | 42 | 0.18 | 48.63 | 41 | 0.19 |
| WHR | Shungin *et al.* GWAS ^2^ | 213.04 | 175 | 0.03 | 209.24 | 174 | 0.04 |
| T2D | Vujkovic *et al.* GWAS ^3^ | 328.24 | 253 | <0.01 | 328.21 | 252 | <0.01 |
| HbA_1c_ | Wheeler *et al.* GWAS ^4^ | 40.40 | 36 | 0.28 | 39.35 | 35 | 0.28 |
| FG | Lagou *et al.* GWAS ^5^ | 24.00 | 27 | 0.63 | 23.98 | 26 | 0.58 |
| FI | Lagou *et al.* GWAS ^5^ | 32.87 | 16 | <0.01 | 31.63 | 15 | <0.01 |
| SBP | Evangelou *et al.* GWAS ^6^ | 95.79 | 82 | 0.15 | 94.81 | 82 | 0.14 |
| DBP | Evangelou *et al.* GWAS ^6^ | 95.82 | 63 | <0.01 | 95.22 | 62 | <0.01 |

Abbreviations: Q, Q-statistic; df, degrees of freedom; P, p-value; BMI, body mass index; WC, waist circumference; WHR, waist-hip ratio; T2D, type 2 diabetes mellitus; HbA_1c_, glycated haemoglobin; FG, fasting glucose; FI, fasting insulin; SBP, systolic blood pressure; DBP, diastolic blood pressure.

**Supplementary file 2, Table 2C.** Assessing directional pleiotropy through MR Egger intercept for metabolic disorder analysis.

| **Exposure** | **Exposure dataset** | **N SNPs** | **Estimate** | **SE** | **P** |
| --- | --- | --- | --- | --- | --- |
| BMI | Pulit *et al.* GWAS ^1^ | 272 | 0.006 | 0.005 | 0.21 |
| WC | Pulit *et al.* GWAS ^1^ | 43 | 0.016 | 0.013 | 0.24 |
| WHR | Shungin *et al.* GWAS ^2^ | 176 | -0.011 | 0.006 | 0.08 |
| T2D | Vujkovic *et al.* GWAS ^3^ | 254 | 0.001 | 0.004 | 0.88 |
| HbA_1c_ | Wheeler *et al.* GWAS ^4^ | 37 | 0.009 | 0.010 | 0.34 |
| FG | Lagou *et al.* GWAS ^5^ | 28 | -0.001 | 0.011 | 0.90 |
| FI | Lagou *et al.* GWAS ^5^ | 17 | 0.032 | 0.042 | 0.46 |
| SBP | Evangelou *et al.* GWAS ^6^ | 83 | -0.014 | 0.016 | 0.36 |
| DBP | Evangelou *et al.* GWAS ^6^ | 64 | -0.010 | 0.015 | 0.54 |

Abbreviations: SE, standard error; P, p-value; BMI, body mass index; WC, waist circumference; WHR, waist-hip ratio; T2D, type 2 diabetes mellitus; HbA_1c_, glycated haemoglobin; FG, fasting glucose; FI, fasting insulin; SBP, systolic blood pressure; DBP, diastolic blood pressure.

**Supplementary file 2, Table 2D.** MR-PRESSO outliers detected results in the analysis of metabolic disorders on combined oral and oropharyngeal cancer risk.

| **Exposure** | **SNP** | **Q statistic** | **P value** | **Q sum** | **Q difference** |
| --- | --- | --- | --- | --- | --- |
| BMI | rs1928295 | 9.75 | <0.001 | 9.75 | -301.70 |
|  | rs17201143 | 9.26 | <0.001 | 19.01 | -292.44 |
|  | rs1320251 | 8.17 | <0.001 | 27.18 | -284.27 |
|  | rs13107325 | 6.38 | 0.01 | 33.56 | -277.89 |
|  | rs3808477 | 6.12 | 0.01 | 39.68 | -271.77 |
|  | rs12140153 | 5.82 | 0.02 | 45.50 | -265.94 |
|  | rs194809 | 5.80 | 0.02 | 51.30 | -260.15 |
|  | rs11655587 | 5.65 | 0.02 | 56.96 | -254.49 |
|  | rs12072739 | 5.57 | 0.02 | 62.53 | -248.92 |
|  | rs12321904 | 5.11 | 0.02 | 67.64 | -243.81 |
|  | rs5215 | 4.99 | 0.03 | 72.63 | -238.82 |
|  | rs2228213 | 4.85 | 0.03 | 77.48 | -233.97 |
|  | rs12282785 | 4.73 | 0.03 | 82.21 | -229.24 |
|  | rs10929925 | 4.34 | 0.04 | 86.55 | -224.90 |
|  | rs2862996 | 4.28 | 0.04 | 90.82 | -220.63 |
|  | rs13021737 | 4.02 | 0.04 | 94.85 | -216.60 |
|  | rs561136 | 4.01 | 0.05 | 98.86 | -212.59 |
|  | rs11047132 | 3.95 | 0.05 | 102.81 | -208.64 |
|  | rs7713317 | 3.90 | 0.05 | 106.71 | -204.74 |
| WC | rs1928295 | 10.74 | <0.001 | 10.74 | -39.53 |
|  | rs806794 | 7.42 | 0.01 | 18.16 | -32.11 |
| WHR | rs12777288 | 12.17 | <0.001 | 12.17 | -200.87 |
|  | rs7213608 | 8.21 | <0.001 | 20.38 | -192.66 |
|  | rs4395620 | 7.34 | 0.01 | 27.72 | -185.32 |
|  | rs2167750 | 6.78 | 0.01 | 34.51 | -178.53 |
|  | rs55747707 | 6.25 | 0.01 | 40.75 | -172.29 |
|  | rs12140153 | 6.16 | 0.01 | 46.92 | -166.13 |
|  | rs12608504 | 5.59 | 0.02 | 52.51 | -160.53 |
|  | rs76699125 | 5.59 | 0.02 | 58.10 | -154.95 |
|  | rs1787013 | 5.05 | 0.02 | 63.14 | -149.90 |
|  | rs6688233 | 5.00 | 0.03 | 68.14 | -144.90 |
|  | rs861029 | 4.93 | 0.03 | 73.07 | -139.98 |
|  | rs17738166 | 4.34 | 0.04 | 77.41 | -135.63 |
| T2D | rs6011155 | 17.92 | <0.01 | 17.92 | -310.28 |
|  | rs12789028 | 8.23 | <0.01 | 26.15 | -302.05 |
|  | rs4833687 | 7.10 | 0.01 | 33.24 | -294.95 |
|  | rs7483027 | 6.54 | 0.01 | 39.78 | -288.42 |
|  | rs10808671 | 6.49 | 0.01 | 46.27 | -281.93 |
|  | rs2269247 | 6.06 | 0.01 | 52.32 | -275.87 |
|  | rs3753693 | 5.92 | 0.01 | 58.25 | -269.95 |
|  | rs1470560 | 5.88 | 0.02 | 64.13 | -264.07 |
|  | rs11759026 | 5.67 | 0.02 | 69.80 | -258.40 |
|  | rs12918782 | 5.47 | 0.02 | 75.27 | -252.93 |
|  | rs174541 | 5.46 | 0.02 | 80.73 | -247.47 |
|  | rs4671799 | 5.20 | 0.02 | 85.93 | -242.27 |
|  | rs3130931 | 5.11 | 0.02 | 91.04 | -237.16 |
|  | rs7664347 | 4.35 | 0.04 | 95.38 | -232.81 |
|  | rs61817176 | 4.30 | 0.04 | 99.69 | -228.51 |
|  | rs13059382 | 4.25 | 0.04 | 103.94 | -224.26 |
|  | rs429358 | 4.23 | 0.04 | 108.17 | -220.03 |
|  | rs60384372 | 4.21 | 0.04 | 112.38 | -215.82 |
|  | rs3744347 | 4.19 | 0.04 | 116.57 | -211.62 |
|  | rs73121277 | 4.11 | 0.04 | 120.69 | -207.51 |
|  | rs757110 | 3.93 | 0.05 | 124.61 | -203.58 |
|  | rs6976111 | 3.93 | 0.05 | 128.54 | -199.66 |
|  | rs10916780 | 3.92 | 0.05 | 132.46 | -195.74 |
| HbA_1c_ | rs4783565 | 11.33 | <0.001 | 11.33 | -29.01 |
|  | rs10774625 | 4.72 | 0.03 | 16.05 | -24.29 |
|  | rs11248914 | 4.01 | 0.05 | 20.06 | -20.29 |
|  | rs17747324 | 3.96 | 0.05 | 24.01 | -16.33 |
| FG | rs983309 | 4.41 | 0.04 | 4.41 | -19.59 |
| FI | rs1167800 | 6.01 | 0.01 | 6.01 | -26.84 |
|  | rs35767 | 5.12 | 0.02 | 11.14 | -21.71 |
|  | rs3822072 | 5.10 | 0.02 | 16.23 | -16.62 |
| SBP | rs2613765 | 7.93 | <0.001 | 7.93 | -87.86 |
|  | rs10069690 | 7.64 | 0.01 | 15.57 | -80.22 |
|  | rs7187540 | 6.59 | 0.01 | 22.16 | -73.63 |
|  | rs260508 | 4.87 | 0.03 | 27.03 | -68.76 |
|  | rs4651224 | 3.95 | 0.05 | 30.98 | -64.81 |
| DBP | rs360153 | 9.71 | <0.001 | 9.71 | -85.95 |
|  | rs11026586 | 5.61 | 0.02 | 15.32 | -80.34 |
|  | rs1607644 | 5.60 | 0.02 | 20.92 | -74.74 |
|  | rs4411245 | 5.27 | 0.02 | 26.19 | -69.47 |
|  | rs61892344 | 4.36 | 0.04 | 30.55 | -65.11 |
|  | rs954767 | 4.11 | 0.04 | 34.67 | -60.99 |
|  | rs1718845 | 3.94 | 0.05 | 38.61 | -57.05 |

Abbreviations: Q-stat, Cochran’s Q statistic; BMI, body mass index; WC, waist circumference; WHR, waist-hip ratio; T2D, type 2 diabetes mellitus; HbA_1c_, glycated haemoglobin; FG, fasting glucose; FI, fasting insulin; SBP, systolic blood pressure; DBP, diastolic blood pressure.

**Supplementary file 2, Table 2E.** MR-PRESSO results for metabolic disorders on combined oral and oropharyngeal cancer.

| **Exposure** | **Outcome** | **Global test RSSobs** | **P-value** | **Outlier test** | **P-value** |
| --- | --- | --- | --- | --- | --- |
| BMI | Oral and oropharyngeal cancer | 314.14 | 0.05 | <0.01 | 1.00 |
| WC | Oral and oropharyngeal cancer | 52.43 | 0.22 | NA | NA |
| WHR | Oral and oropharyngeal cancer | 215.50 | 0.04 | <0.01 | 1.00 |
| T2D | Oral and oropharyngeal cancer | 330.88 | <0.01 | <0.01 | 1.00 |
| HbA_1c_ | Oral and oropharyngeal cancer | 41.66 | 0.33 | NA | NA |
| FG | Oral and oropharyngeal cancer | 25.34 | 0.68 | NA | NA |
| FI | Oral and oropharyngeal cancer | 38.87 | 0.01 | 1.42E-05 | 1.00 |
| SBP | Oral and oropharyngeal cancer | 98.16 | 0.15 | NA | NA |
| DBP | Oral and oropharyngeal cancer | 99.40 | <0.01 | <0.01 | 1.00 |

Abbreviations: SE, standard error; P, p-value; BMI, body mass index; WC, waist circumference; WHR, waist-hip ratio; T2D, type 2 diabetes mellitus; HbA_1c_, glycated haemoglobin; FG, fasting glucose; FI, fasting insulin; SBP, systolic blood pressure; DBP, diastolic blood pressure.

**Supplementary file 2, Table 2F.** Outlier corrected results in the analysis of metabolic disorders on combined oral and oropharyngeal cancer risk.

| **Exposure** | **N SNP** | **Method** | **Beta** | **SE** | **OR** | **CIL** | **CIU** | **P value** |
| --- | --- | --- | --- | --- | --- | --- | --- | --- |
| BMI | 253 | IVW | -0.27 | 0.10 | 0.77 | 0.62 | 0.94 | 0.01 |
|  | 253 | MR Egger | -0.39 | 0.26 | 0.68 | 0.41 | 1.13 | 0.13 |
|  | 253 | Weighted median | -0.41 | 0.17 | 0.67 | 0.48 | 0.93 | 0.02 |
|  | 253 | Weighted mode | -0.49 | 0.25 | 0.61 | 0.37 | 1.00 | 0.05 |
| WC | 41 | IVW | -0.43 | 0.16 | 0.65 | 0.47 | 0.89 | 0.01 |
|  | 41 | MR Egger | -0.6 | 0.44 | 0.55 | 0.23 | 1.31 | 0.18 |
|  | 41 | Weighted median | -0.44 | 0.24 | 0.64 | 0.40 | 1.04 | 0.07 |
|  | 41 | Weighted mode | -0.4 | 0.31 | 0.67 | 0.36 | 1.24 | 0.21 |
| WHR | 164 | IVW | -0.13 | 0.13 | 0.87 | 0.68 | 1.13 | 0.30 |
|  | 164 | MR Egger | 0.5 | 0.34 | 1.65 | 0.84 | 3.24 | 0.15 |
|  | 164 | Weighted median | -0.04 | 0.21 | 0.96 | 0.64 | 1.45 | 0.86 |
|  | 164 | Weighted mode | -0.06 | 0.41 | 0.94 | 0.43 | 2.09 | 0.88 |
| T2D | 231 | IVW | -0.09 | 0.04 | 0.91 | 0.84 | 0.99 | 0.03 |
|  | 231 | MR Egger | -0.04 | 0.08 | 0.96 | 0.82 | 1.12 | 0.60 |
|  | 231 | Weighted median | -0.16 | 0.07 | 0.85 | 0.74 | 0.97 | 0.02 |
|  | 231 | Weighted mode | -0.2 | 0.1 | 0.82 | 0.67 | 0.99 | 0.04 |
| HbA_1c_ | 33 | IVW | -0.55 | 0.29 | 0.58 | 0.33 | 1.01 | 0.05 |
|  | 33 | MR Egger | -0.75 | 0.51 | 0.47 | 0.17 | 1.30 | 0.16 |
|  | 33 | Weighted median | -0.65 | 0.43 | 0.52 | 0.23 | 1.22 | 0.13 |
|  | 33 | Weighted mode | -0.63 | 0.45 | 0.53 | 0.22 | 1.29 | 0.17 |
| FG | 27 | IVW | 0.14 | 0.23 | 1.15 | 0.73 | 1.80 | 0.55 |
|  | 27 | MR Egger | 0.18 | 0.43 | 1.19 | 0.52 | 2.75 | 0.68 |
|  | 27 | Weighted median | 0.18 | 0.33 | 1.2 | 0.63 | 2.28 | 0.59 |
|  | 27 | Weighted mode | 0.12 | 0.31 | 1.12 | 0.61 | 2.06 | 0.71 |
| FI | 14 | IVW | -0.97 | 0.51 | 0.38 | 0.14 | 1.02 | 0.06 |
|  | 14 | MR Egger | -0.86 | 2.47 | 0.42 | 0.01 | 53.86 | 0.73 |
|  | 14 | Weighted median | -0.4 | 0.74 | 0.67 | 0.16 | 2.83 | 0.59 |
|  | 14 | Weighted mode | -2.88 | 1.68 | 0.06 | 0.01 | 1.50 | 0.11 |
| SBP | 78 | IVW | -0.01 | 0.02 | 0.99 | 0.96 | 1.02 | 0.46 |
|  | 78 | MR Egger | -0.01 | 0.07 | 0.99 | 0.87 | 1.14 | 0.94 |
|  | 78 | Weighted median | -0.02 | 0.02 | 0.98 | 0.94 | 1.03 | 0.49 |
|  | 78 | Weighted mode | -0.02 | 0.05 | 0.98 | 0.88 | 1.08 | 0.63 |
| DBP | 57 | IVW | -0.06 | 0.03 | 0.94 | 0.89 | 1.00 | 0.06 |
|  | 57 | MR Egger | -0.02 | 0.1 | 0.98 | 0.81 | 1.19 | 0.83 |
|  | 57 | Weighted median | -0.05 | 0.05 | 0.96 | 0.87 | 1.05 | 0.32 |
|  | 57 | Weighted mode | 0.12 | 0.11 | 1.12 | 0.91 | 1.40 | 0.29 |

Abbreviations: SE, standard error; OR, odds ratio; CI, confidence intervals; IVW, inverse variance weighted; BMI, body mass index; WC, waist circumference; WHR, waist-hip ratio; T2D, type 2 diabetes mellitus; HbA_1c_, glycated haemoglobin; FG, fasting glucose; FI, fasting insulin; SBP, systolic blood pressure; DBP, diastolic blood pressure.

**Supplementary file 2, Table 2G.** Assessing violation of the NO Measurement Error (NOME) assumption for instruments used in MR-Egger regression.

| **Exposure** | **Exposure dataset** | **I^2^ unweighted** | **I^2^ weighted** |
| --- | --- | --- | --- |
| BMI | Pulit *et al.* GWAS ^1^ | 0.94 | 0.93 |
| WC | Pulit *et al.* GWAS ^1^ | 0.89 | 0.87 |
| WHR | Shungin *et al.* GWAS ^2^ | 0.90 | 0.88 |
| T2D | Vujkovic *et al.* GWAS ^3^ | 0.97 | 0.97 |
| HbA_1c_ | Wheeler *et al.* GWAS ^4^ | 0.94 | 0.92 |
| FG | Lagou *et al.* GWAS ^5^ | 0.98 | 0.98 |
| FI | Lagou *et al.* GWAS ^5^ | 0.55 | 0.37 |
| SBP | Evangelou *et al.* GWAS ^6^ | 0.72 | 0.35 |
| DBP | Evangelou *et al.* GWAS ^6^ | 0.81 | 0.59 |

Abbreviations: I^2^, I-squared statistic; BMI, body mass index; WC, waist circumference; WHR, waist-hip ratio; T2D, type 2 diabetes mellitus; HbA_1c_, glycated haemoglobin; FG, fasting glucose; FI, fasting insulin; SBP, systolic blood pressure; DBP, diastolic blood pressure.

**Supplementary file 2, Table 2H.** SIMEX correction MR Egger regression results for where NO Measurement Error (NOME) assumption may have been violated (I^2^ <0.90).

| **Exposure** | **Outcome** | **OR** | **CIL** | **CIU** | **P-value** |
| --- | --- | --- | --- | --- | --- |
| WC | Oral and oropharyngeal cancer | 0.64 | 0.25 | 1.63 | 0.36 |
| FI | Oral and oropharyngeal cancer | 0.01 | 2.10E-05 | 4.35 | 0.16 |
| SBP | Oral and oropharyngeal cancer | 1.15 | 1.05 | 1.26 | <0.01 |
| DBP | Oral and oropharyngeal cancer | 1.07 | 0.91 | 1.25 | 0.43 |

Abbreviations: OR, odds ratio; CI, confidence intervals; WC, waist circumference; FI, fasting insulin; SBP, systolic blood pressure; DBP, diastolic blood pressure.

**Supplementary file 2, Table 2I.** Mendelian randomization results evaluating instrument-risk factor effects.

|  |  | | | **IVW** | | **Weighted median** | | **Weighted mode** | | **MR-Egger** | |
| --- | --- | --- | --- | --- | --- | --- | --- | --- | --- | --- | --- |
| **Exposure** | **Outcome** | **Exposure/**  **Outcome**  **source** | **N SNPs** | **Beta (SE)** | **P** | **Beta (SE)** | **P** | **Beta (SE)** | **P** | **Beta (SE)** | **P** |
| BMI | Smoking initiation | Pulit *et al.* GWAS ^1^/ Liu et al. GWAS ^7^ | 273 | 0.21 (0.03) | <0.001 | 0.14 (0.03) | <0.001 | 0.08 (0.04) | 0.07 | 0.07 (0.06) | 0.27 |
|  | Comprehensive smoking index | Pulit *et al.* GWAS ^1^/ Wootton et al.^8^ | 432 | 0.10 (0.01) | <0.001 | 0.08 (0.01) | <0.001 | 0.06 (0.02) | 0.01 | 0.09 (0.02) | <0.001 |
|  | Alcohol drinks per week | Pulit *et al.* GWAS ^1^/ Liu et al. GWAS ^7^ | 274 | -0.04 (0.01) | 0.01 | -0.05 (0.01) | <0.001 | -0.10 (0.02) | <0.001 | -0.11 (0.03) | <0.001 |
|  | Risk tolerance | Pulit *et al.* GWAS ^1^/ Karlsson Linner et al. GWAS ^9^ | 301 | 0.04 (0.01) | <0.001 | 0.001 (0.02) | 0.92 | -0.06 (0.03) | 0.05 | -0.03 (0.03) | 0.41 |
|  | Educational attainment | Pulit *et al.* GWAS ^1^/ Lee et al. GWAS ^10^ | 299 | -0.16 (0.02) | <0.001 | -0.11 (0.01) | <0.001 | -0.03 (0.03) | 0.47 | 0.01 (0.04) | 0.82 |
| WC | Smoking initiation | Pulit *et al.* GWAS ^1^/ Liu et al. GWAS ^7^ | 43 | 0.21 (0.05) | <0.001 | 0.09 (0.04) | 0.03 | 0.05 (0.04) | 0.25 | -0.13 (0.14) | 0.35 |
|  | Comprehensive smoking index | Pulit *et al.* GWAS ^1^/ Wootton et al.^8^ | 45 | 0.10 (0.02) | <0.001 | 0.05 (0.02) | 0.01 | 0.01 (0.02) | 0.88 | -0.02 (0.06) | 0.77 |
|  | Alcohol drinks per week | Pulit *et al.* GWAS ^1^/ Liu et al. GWAS ^7^ | 43 | -0.09 (0.02) | <0.001 | -0.11 (0.02) | <0.001 | -0.12 (0.02) | <0.001 | -0.20 (0.06) | <0.001 |
|  | Risk tolerance | Pulit *et al.* GWAS ^1^/ Karlsson Linner et al. GWAS ^9^ | 45 | 0.01 (0.02) | 0.71 | -0.04 (0.02) | 0.05 | -0.05 (0.03) | 0.08 | -0.07 (0.06) | 0.27 |
|  | Educational attainment | Pulit *et al.* GWAS ^1^/ Lee et al. GWAS ^10^ | 45 | -0.06 (0.03) | 0.06 | -0.06 (0.02) | 0.01 | 0.01 (0.02) | 0.77 | 0.08 (0.08) | 0.34 |
| WHR | Smoking initiation | Shungin *et al.* GWAS ^2^/ Liu et al. GWAS ^7^ | 174 | 0.18 (0.03) | <0.001 | 0.09 (0.03) | <0.001 | 0.07 (0.04) | 0.12 | 0.02 (0.09) | 0.84 |
|  | Comprehensive smoking index | Pulit *et al.* GWAS ^1^/ Wootton et al.^8^ | 199 | 0.09 (0.01) | <0.001 | 0.04 (0.01) | <0.001 | 0.01 (0.02) | 0.80 | -0.02 (0.03) | 0.47 |
|  | Alcohol drinks per week | Shungin *et al.* GWAS ^2^/ Liu et al. GWAS ^7^ | 174 | -0.03 (0.02) | 0.11 | -0.02 (0.02) | 0.21 | -0.02 (0.02) | 0.30 | -0.07 (0.04) | 0.11 |
|  | Risk tolerance | Shungin *et al.* GWAS ^2^/ Karlsson Linner et al. GWAS ^9^ | 199 | 0.04 (0.02) | 0.02 | -0.001 (0.02) | 0.96 | -0.04 (0.03) | 0.18 | -0.04 (0.04) | 0.32 |
|  | Educational attainment | Shungin *et al.* GWAS ^2^/ Lee et al. GWAS ^10^ | 196 | -0.11 (0.02) | <0.001 | -0.03 (0.02) | 0.10 | 0.02 (0.02) | 0.40 | 0.08 (0.06) | 0.15 |
| T2D | Smoking initiation | Vujkovic *et al.* GWAS ^3^/ Liu et al. GWAS ^7^ | 255 | 0.02 (0.01) | 0.12 | 0.02 (0.01) | 0.08 | 0.02 (0.01) | 0.08 | -0.01 (0.02) | 0.68 |
|  | Comprehensive smoking index | Pulit *et al.* GWAS ^1^/ Wootton et al.^8^ | 273 | 0.01 (0.004) | 0.004 | 0.003 (0.004) | 0.48 | 0.001 (0.004) | 0.75 | -0.01 (0.008) | 0.13 |
|  | Alcohol drinks per week | Vujkovic *et al.* GWAS ^3^/ Liu et al. GWAS ^7^ | 257 | -0.02 (0.01) | <0.001 | -0.02 (0.01) | <0.001 | -0.02 (0.01) | <0.001 | -0.02 (0.01) | 0.11 |
|  | Risk tolerance | Vujkovic *et al.* GWAS ^3^/ Karlsson Linner et al. GWAS ^9^ | 274 | -0.0002 (0.004) | 0.96 | -0.01 (0.01) | 0.38 | -0.01 (0.01) | 0.30 | -0.01 (0.01) | 0.15 |
|  | Educational attainment | Vujkovic *et al.* GWAS ^3^/ Lee et al. GWAS ^10^ | 272 | -0.02 (0.01) | 0.01 | -0.01 (0.01) | 0.11 | -0.0001 (0.004) | 0.97 | 0.02 (0.01) | 0.10 |
| HbA_1c_ | Smoking initiation | Wheeler *et al.* GWAS ^4^/ Liu et al. GWAS ^7^ | 37 | 0.03 (0.06) | 0.64 | 0.001 (0.06) | 0.99 | 0.002 (0.05) | 0.98 | 0.11 (0.10) | 0.30 |
|  | Comprehensive smoking index | Pulit *et al.* GWAS ^1^/ Wootton et al.^8^ | 40 | 0.03 (0.02) | 0.19 | 0.01 (0.02) | 0.73 | 0.01 (0.03) | 0.83 | 0.05 (0.04) | 0.29 |
|  | Alcohol drinks per week | Wheeler *et al.* GWAS ^4^/ Liu et al. GWAS ^7^ | 37 | 0.01 (0.03) | 0.69 | 0.05 (0.03) | 0.12 | 0.08 (0.03) | 0.02 | 0.11 (0.05) | 0.04 |
|  | Risk tolerance | Wheeler *et al.* GWAS ^4^/ Karlsson Linner et al. GWAS ^9^ | 40 | -0.001 (0.02) | 0.97 | 0.01 (0.03) | 0.68 | 0.01 (0.03) | 0.69 | 0.003 (0.04) | 0.95 |
|  | Educational attainment | Wheeler *et al.* GWAS ^4^/ Lee et al. GWAS ^10^ | 40 | -0.01 (0.04) | 0.80 | -0.02 (0.03) | 0.56 | 0.04 (0.04) | 0.30 | -0.001 (0.08) | 0.99 |
| DBP | Smoking initiation | Evangelou *et al.* GWAS ^6^/ Liu et al. GWAS ^7^ | 64 | -0.01 (0.01) | 0.24 | -0.0004 (0.01) | 0.95 | 0.01 (0.01) | 0.45 | 0.004 (0.02) | 0.85 |
|  | Comprehensive smoking index | Pulit *et al.* GWAS ^1^/ Wootton et al.^8^ | 75 | -0.003 (0.002) | 0.99 | -0.002 (0.002) | 0.99 | -0.003 (0.004) | 0.99 | -0.002 (0.007) | 0.99 |
|  | Alcohol drinks per week | Evangelou *et al.* GWAS ^6^/ Liu et al. GWAS ^7^ | 64 | 0.0004 (0.004) | 0.91 | 0.001 (0.003) | 0.79 | 0.005 (0.01) | 0.44 | -0.003 (0.01) | 0.77 |
|  | Risk tolerance | Evangelou *et al.* GWAS ^6^/ Karlsson Linner et al. GWAS ^9^ | 75 | 0.002 (0.003) | 0.53 | 0.002 (0.003) | 0.53 | 0.004 (0.01) | 0.64 | 0.017 (0.01) | 0.05 |
|  | Educational attainment | Evangelou *et al.* GWAS ^6^/ Lee et al. GWAS ^10^ | 74 | 0.001 (0.004) | 0.75 | -0.001 (0.003) | 0.67 | -0.01 (0.008) | 0.11 | 0.02 (0.01) | 0.06 |

Abbreviations: IVW, inverse variance weighted; OR, odds ratio; CI, confidence intervals; P, p-value; BMI, body mass index; WC, waist circumference; WHR, waist-hip ratio; T2D, type 2 diabetes mellitus;, glycated haemoglobin; DBP, diastolic blood pressure. OR are expressed per 1 standard deviation (SD) increase in genetically predicted BMI (4.81 kg/m2), WC (0.09 unit), WHR (0.10 unit), T2D (1-log unit higher odds of T2D), HbA1c (1-log-unit % higher glycated haemoglobin), and DBP (1 unit mmHg increase). Outcome beta estimates reflect the standard deviation of the phenotype.

**Supplementary file 2, Table 2J.** Assessing heterogeneity in Mendelian randomization results evaluating instrument-risk factor effects.

| **Exposure** | **Outcome** | **Q IVW** | **df** | **P** | **Q MR Egger** | **df** | **P** |
| --- | --- | --- | --- | --- | --- | --- | --- |
| BMI | Smoking initiation | 1033.81 | 272 | <0.001 | 1011.43 | 271 | <0.001 |
|  | Comprehensive smoking index | 1721.73 | 431 | <0.001 | 1720.93 | 430 | <0.001 |
|  | Alcohol drinks per week | 952.47 | 273 | <0.001 | 928.23 | 272 | <0.001 |
|  | Risk tolerance | 831.50 | 300 | <0.001 | 813.38 | 299 | <0.001 |
|  | Educational attainment | 1939.06 | 298 | <0.001 | 1798.21 | 297 | <0.001 |
| WC | Smoking initiation | 271.19 | 42 | <0.001 | 231.18 | 41 | <0.001 |
|  | Comprehensive smoking index | 352.22 | 44 | <0.001 | 320.82 | 43 | <0.001 |
|  | Alcohol drinks per week | 177.18 | 42 | <0.001 | 163.17 | 41 | <0.001 |
|  | Risk tolerance | 155.99 | 44 | <0.001 | 149.70 | 43 | <0.001 |
|  | Educational attainment | 471.72 | 44 | <0.001 | 437.88 | 43 | <0.001 |
| WHR | Smoking initiation | 719.07 | 173 | <0.001 | 702.72 | 172 | <0.001 |
|  | Comprehensive smoking index | 770.04 | 198 | <0.001 | 721.88 | 197 | <0.001 |
|  | Alcohol drinks per week | 563.69 | 173 | <0.001 | 560.08 | 172 | <0.001 |
|  | Risk tolerance | 496.94 | 198 | <0.001 | 486.63 | 197 | <0.001 |
|  | Educational attainment | 1429.33 | 195 | <0.001 | 1335.57 | 194 | <0.001 |
| T2D | Smoking initiation | 852.86 | 254 | <0.001 | 845.63 | 253 | <0.001 |
|  | Comprehensive smoking index | 1135.72 | 272 | <0.001 | 1084.57 | 271 | <0.001 |
|  | Alcohol drinks per week | 852.40 | 256 | <0.001 | 850.76 | 255 | <0.001 |
|  | Risk tolerance | 556.93 | 273 | <0.001 | 551.33 | 272 | <0.001 |
|  | Educational attainment | 1893.23 | 271 | <0.001 | 1804.42 | 270 | <0.001 |
| HbA_1c_ | Smoking initiation | 93.86 | 36 | <0.001 | 91.43 | 35 | <0.001 |
|  | Comprehensive smoking index | 111.96 | 39 | <0.001 | 111.40 | 38 | <0.001 |
|  | Alcohol drinks per week | 83.35 | 36 | <0.001 | 72.02 | 35 | <0.001 |
|  | Risk tolerance | 48.21 | 39 | 0.15 | 48.20 | 38 | 0.12 |
|  | Educational attainment | 249.64 | 39 | <0.001 | 249.48 | 38 | <0.001 |
| DBP | Smoking initiation | 207.72 | 63 | <0.001 | 206.47 | 62 | <0.001 |
|  | Comprehensive smoking index | 203.79 | 74 | <0.001 | 203.75 | 73 | <0.001 |
|  | Alcohol drinks per week | 213.03 | 63 | <0.001 | 212.61 | 62 | <0.001 |
|  | Risk tolerance | 120.47 | 74 | <0.001 | 114.97 | 73 | <0.001 |
|  | Educational attainment | 308.47 | 73 | <0.001 | 293.92 | 72 | <0.001 |

Abbreviations: Q, Q-statistic; df, degrees of freedom; P, p-value; BMI, body mass index; WC, waist circumference; WHR, waist-hip ratio; T2D, type 2 diabetes mellitus; HbA_1c_, glycated haemoglobin; DBP, diastolic blood pressure.

**Supplementary file 2, Table 2K.** Assessing directional pleiotropy in Mendelian randomization results evaluating instrument-risk factor effects.

| **Exposure** | **Outcome** | **MR Egger intercept estimate** | **SE** | **P** |
| --- | --- | --- | --- | --- |
| BMI | Smoking initiation | 0.003 | 0.001 | 0.01 |
|  | Comprehensive smoking index | 0.001 | 0.003 | 0.05 |
|  | Alcohol drinks per week | 0.002 | 0.001 | 0.01 |
|  | Risk tolerance | 0.001 | 0.001 | 0.02 |
|  | Educational attainment | -0.003 | 0.001 | <0.001 |
| WC | Smoking initiation | 0.010 | 0.004 | 0.01 |
|  | Comprehensive smoking index | 0.003 | 0.002 | 0.04 |
|  | Alcohol drinks per week | 0.003 | 0.001 | 0.07 |
|  | Risk tolerance | 0.002 | 0.002 | 0.19 |
|  | Educational attainment | -0.004 | 0.002 | 0.08 |
| WHR | Smoking initiation | 0.003 | 0.002 | 0.05 |
|  | Comprehensive smoking index | 0.002 | 0.001 | <0.001 |
|  | Alcohol drinks per week | 0.001 | 0.001 | 0.29 |
|  | Risk tolerance | 0.001 | 0.001 | 0.04 |
|  | Educational attainment | -0.004 | 0.001 | <0.001 |
| T2D | Smoking initiation | 0.001 | 0.001 | 0.14 |
|  | Comprehensive smoking index | 0.001 | 0.001 | <0.001 |
|  | Alcohol drinks per week | -0.001 | 0.001 | 0.48 |
|  | Risk tolerance | 0.001 | 0.001 | 0.10 |
|  | Educational attainment | -0.002 | 0.001 | <0.001 |
| HbA_1c_ | Smoking initiation | -0.002 | 0.002 | 0.34 |
|  | Comprehensive smoking index | -0.0003 | 0.001 | 0.66 |
|  | Alcohol drinks per week | -0.002 | 0.001 | 0.02 |
|  | Risk tolerance | 0.001 | 0.001 | 0.92 |
|  | Educational attainment | -0.001 | 0.001 | 0.88 |
| DBP | Smoking initiation | -0.002 | 0.003 | 0.54 |
|  | Comprehensive smoking index | -0.001 | 0.001 | 0.90 |
|  | Alcohol drinks per week | 0.001 | 0.002 | 0.73 |
|  | Risk tolerance | -0.002 | 0.001 | 0.07 |
|  | Educational attainment | -0.003 | 0.002 | 0.06 |

Abbreviations: SE, standard error; P, p-value; BMI, body mass index; WC, waist circumference; WHR, waist-hip ratio; T2D, type 2 diabetes mellitus; HbA_1c_, glycated haemoglobin; DBP, diastolic blood pressure.

**Supplementary file 2, Table 2L.** Outlier corrected Mendelian randomization results evaluating instrument-risk factor effects.

| **Exposure** | **Outcome** | **N Outlier SNPs** | **IVW Beta** | **SE** | **P** |
| --- | --- | --- | --- | --- | --- |
| BMI | Smoking initiation | 64 | 0.23 | 0.02 | <0.001 |
|  | Comprehensive smoking index | 132 | 0.10 | 0.01 | <0.001 |
|  | Alcohol drinks per week | 80 | -0.01 | 0.01 | 0.41 |
|  | Risk tolerance | 74 | 0.04 | 0.01 | <0.001 |
|  | Educational attainment | 99 | -0.16 | 0.01 | <0.001 |
| WC | Smoking initiation | 24 | 0.17 | 0.04 | <0.001 |
|  | Comprehensive smoking index | 26 | 0.09 | 0.02 | <0.001 |
|  | Alcohol drinks per week | 14 | -0.09 | 0.01 | <0.001 |
|  | Risk tolerance | 11 | 0.01 | 0.02 | 0.50 |
|  | Educational attainment | 19 | -0.06 | 0.02 | <0.001 |
| WHR | Smoking initiation | 52 | 0.15 | 0.02 | <0.001 |
|  | Comprehensive smoking index | 51 | 0.08 | 0.01 | <0.001 |
|  | Alcohol drinks per week | 39 | -0.02 | 0.01 | 0.12 |
|  | Risk tolerance | 36 | 0.02 | 0.01 | 0.02 |
|  | Educational attainment | 75 | -0.11 | 0.01 | <0.001 |
| T2D | Smoking initiation | 66 | 0.01 | 0.01 | 0.01 |
|  | Comprehensive smoking index | 79 | 0.01 | 0.002 | <0.001 |
|  | Alcohol drinks per week | 49 | -0.02 | 0.01 | <0.001 |
|  | Risk tolerance | 42 | 0.01 | 0.01 | 0.30 |
|  | Educational attainment | 101 | -0.01 | 0.01 | <0.001 |
| HbA_1c_ | Smoking initiation | 6 | 0.02 | 0.04 | 0.64 |
|  | Comprehensive smoking index | 9 | 0.02 | 0.01 | 0.13 |
|  | Alcohol drinks per week | 8 | 0.01 | 0.03 | 0.62 |
|  | Risk tolerance | 1 | 0.01 | 0.02 | 0.96 |
|  | Educational attainment | 11 | -0.07 | 0.02 | <0.001 |
| DBP | Smoking initiation | 13 | 0.01 | 0.01 | 0.91 |
|  | Comprehensive smoking index | 18 | -0.003 | 0.002 | 0.03 |
|  | Alcohol drinks per week | 17 | 0.01 | 0.01 | 0.30 |
|  | Risk tolerance | 9 | 0.01 | 0.01 | 0.39 |
|  | Educational attainment | 25 | 0.01 | 0.01 | 0.66 |

Abbreviations: IVW, inverse variance weighted; OR, odds ratio; CI, confidence intervals; P, p-value; BMI, body mass index; WC, waist circumference; WHR, waist-hip ratio; T2D, type 2 diabetes mellitus;, glycated haemoglobin; DBP, diastolic blood pressure. OR are expressed per 1 standard deviation (SD) increase in genetically predicted BMI (4.81 kg/m2), WC (0.09 unit), WHR (0.10 unit), T2D (1-log unit higher odds of T2D), HbA1c (1-log-unit % higher glycated haemoglobin), and DBP (1 unit mmHg increase). Outcome beta estimates reflect the standard deviation of the phenotype.

**Supplementary references**

1. Pulit SL, Stoneman C, Morris AP, Wood AR, Glastonbury CA, Tyrrell J, et al. Meta-analysis of genome-wide association studies for body fat distribution in 694 649 individuals of European ancestry. *Human Molecular Genetics*. 2019;28(1):166-74.

2. Shungin D, Winkler TW, Croteau-Chonka DC, Ferreira T, Locke AE, Mägi R, et al. New genetic loci link adipose and insulin biology to body fat distribution. *Nature*. 2015;518(7538):187-96.

3. Vujkovic M, Keaton JM, Lynch JA, Miller DR, Zhou J, Tcheandjieu C, et al. Discovery of 318 new risk loci for type 2 diabetes and related vascular outcomes among 1.4 million participants in a multi-ancestry meta-analysis. *Nature Genetics*. 2020;52(7):680-91.

4. Wheeler E, Leong A, Liu CT, Hivert MF, Strawbridge RJ, Podmore C, et al. Impact of common genetic determinants of Hemoglobin A1c on type 2 diabetes risk and diagnosis in ancestrally diverse populations: A transethnic genome-wide meta-analysis. *PLoS Med*. 2017;14(9):e1002383.

5. Lagou V, Mägi R, Hottenga J-J, Grallert H, Perry JRB, Bouatia-Naji N, et al. Sex-dimorphic genetic effects and novel loci for fasting glucose and insulin variability. *Nature Communications*. 2021;12(1):24.

6. Evangelou E, Warren HR, Mosen-Ansorena D, Mifsud B, Pazoki R, Gao H, et al. Genetic analysis of over 1 million people identifies 535 new loci associated with blood pressure traits. *Nature Genetics*. 2018;50(10):1412-25.

7. Liu MZ, Jiang Y, Wedow R, Li Y, Brazel DM, Chen F, et al. Association studies of up to 1.2 million individuals yield new insights into the genetic etiology of tobacco and alcohol use. *Nature Genetics*. 2019;51(2):237.

8. Wootton RE, Richmond RC, Stuijfzand BG, Lawn RB, Sallis HM, Taylor GMJ, et al. Evidence for causal effects of lifetime smoking on risk for depression and schizophrenia: a Mendelian randomisation study. *Psychol Med*. 2019:1-9.

9. Karlsson Linner R, Biroli P, Kong E, Meddens SFW, Wedow R, Fontana MA, et al. Genome-wide association analyses of risk tolerance and risky behaviors in over 1 million individuals identify hundreds of loci and shared genetic influences. *Nature Genetics*. 2019;51(2):245-57.

10. Lee J, Wedow R, Okbay A, Kong E, Maghzian O, Zacher M, et al. Gene discovery and polygenic prediction from a genome-wide association study of educational attainment in 1.1 million individuals. *Nature Genetics*. 2018;50(8):1112-21.
