# Supplementary material for: Evaluating the effect of metabolic traits on oral and oropharyngeal cancer risk using Mendelian randomization
Source: eLife. 2023 Apr 12;12:e82674. doi: 10.7554/eLife.82674 (PMC10147379; doi:10.7554/eLife.82674)
Supplement: Transparent reporting form [file elife-82674-transrepform1.pdf]

eLIFE
1st Floor
24 Hills Road
Cambridge CB2 1JP, UK
P 01223 855340
W elifesciences.org
T @elife

eLife's transparent reporting form

We encourage authors to provide detailed information *within their submission* to facilitate the interpretation and replication of experiments. Authors can upload supporting documentation to indicate the use of appropriate reporting guidelines for health-related research (see EQUATOR Network), life science research (see the BioSharing Information Resource), or the ARRIVE guidelines for reporting work involving animal research. Where applicable, authors should refer to any relevant reporting standards documents in this form.

If you have any questions, please consult our Journal Policies and/or contact us: editorial@elifesciences.org.

Sample-size estimation

- You should state whether an appropriate sample size was computed when the study was being designed
- You should state the statistical method of sample size computation and any required assumptions
- If no explicit power analysis was used, you should describe how you decided what sample (replicate) size (number) to use

Please outline where this information can be found within the submission (e.g., sections or figure legends), or explain why this information doesn't apply to your submission:

Power calculations can be found in Supplementary file 2 - Figures S1A-C. Sample size was calculated using an online calculator for MR available at: https://shiny.cnsgenomics.com/mRnd/. Calculations were performed separately for combined head and neck cancer subsites and stratified by individual oral and oropharyngeal subsite (Supplementary file 2 - Figures S1A-C).

Genetic instruments were estimated to explain between 0.5% (FI) and 4% (BMI) of their respective metabolic trait. In analyses where BMI was the exposure, we had 70% power to detect an association with an OR of 1.2 or more at an α of 0.05 for combined oral and oropharyngeal cancer. Power was lower for other metabolic traits and reduced when stratifying analyses by subsite.

Replicates

- You should report how often each experiment was performed
- You should include a definition of biological versus technical replication
- The data obtained should be provided and sufficient information should be provided to indicate the number of independent biological and/or technical replicates
- If you encountered any outliers, you should describe how these were handled
- Criteria for exclusion/inclusion of data should be clearly stated
- High-throughput sequence data should be uploaded before submission, with a private link for reviewers provided (these are available from both GEO and ArrayExpress)

Please outline where this information can be found within the submission (e.g., sections or figure legends), or explain why this information doesn't apply to your submission:

Replication not applicable as this is a Mendelian randomization study. All GWAS were replicated (in their respective studies), ensuring valid genetic instruments were used for each exposure and outcome trait. Outliers were corrected for/ removed where indicated as described in Methods section (page 17).

Statistical reporting

- Statistical analysis methods should be described and justified
- Raw data should be presented in figures whenever informative to do so (typically when N per group is less than 10)
- For each experiment, you should identify the statistical tests used, exact values of N, definitions of center, methods of multiple test correction, and dispersion and precision measures (e.g., mean, median, SD, SEM, confidence intervals; and, for the major substantive results, a measure of effect size (e.g., Pearson's r, Cohen's d)
- Report exact p-values wherever possible alongside the summary statistics and 95% confidence intervals. These should be reported for all key questions and not only when the p-value is less than 0.05.

Please outline where this information can be found within the submission (e.g., sections or figure legends), or explain why this information doesn't apply to your submission:

Mendelian randomization statistical methods (and sensitivity analyses) are described and justified in pages 8-10. Summary statistics used for metabolic trait exposures are available in Supplementary file 1. Results (and N per group) have been transparently reported in pages 11-19, Table1, Figure 2 and Supplementary file 2- tables and figures.

(For large datasets, or papers with a very large number of statistical tests, you may upload a single table file with tests, Ns, etc., with reference to sections in the manuscript.)

Group allocation

- Indicate how samples were allocated into experimental groups (in the case of clinical studies, please specify allocation to treatment method); if randomization was used, please also state if restricted randomization was applied
- Indicate if masking was used during group allocation, data collection and/or data analysis

Please outline where this information can be found within the submission (e.g., sections or figure legends), or explain why this information doesn't apply to your submission:

Given this is a Mendelian randomization study, we did not allocate individuals into experimental groups. Instead genetic variants (randomized naturally during meiosis) were used as proxies for metabolic trait exposures and risk of head and neck cancer outcomes (cases versus controls from summary-level genome-wide association studies). This method is analgous to a randomized controlled trial.

Additional data files ("source data")

- We encourage you to upload relevant additional data files, such as numerical data that are represented as a graph in a figure, or as a summary table
- Where provided, these should be in the most useful format, and they can be uploaded as "Source data" files linked to a main figure or table
- Include model definition files including the full list of parameters used
- Include code used for data analysis (e.g., R, MatLab)
- Avoid stating that data files are "available upon request"

Please indicate the figures or tables for which source data files have been provided:

A copy of the code and all data files used in this study are available at GitHub (https://github.com/MGormley12/metabolic_trait_hnc_mr.git).
